# New Evaluation of the Electronically Activated Recorder (EAR): Obtrusiveness, Compliance, and Participant Self-selection Effects

**DOI:** 10.3389/fpsyg.2017.00658

**Published:** 2017-04-28

**Authors:** Joseph H. Manson, Megan L. Robbins

**Affiliations:** ^1^Department of Anthropology and Center for Behavior, Evolution, and Culture, University of California, Los Angeles, Los AngelesCA, USA; ^2^Department of Psychology, University of California, Riverside, RiversideCA, USA

**Keywords:** naturalistic observation, experience sampling, Electronically Activated Recorder, HEXACO, conscientiousness

## Abstract

The Electronically Activated Recorder (EAR) is a method for collecting periodic brief audio snippets of participants’ daily lives using a portable recording device. The EAR can potentially intrude into people’s privacy, alter their natural behavior, and introduce self-selection biases greater than in other types of social science methods. Previous research (Mehl and Holleran, 2007, hereafter M&H) has shown that participant non-compliance with, and perceived obtrusiveness of, an EAR protocol are both low. However, these questions have not been addressed in jurisdictions that require the consent of all parties to recording conversations. This EAR study required participants to wear a button bearing a microphone icon and the words “This conversation may be recorded” to comply with California’s all-party consent law. Results revealed self-reported obtrusiveness and non-compliance were actually lower in the present study than in the M&H study. Behaviorally assessed non-compliance did not differ between the two studies. Participants in the present study talked more about being in the study than participants in the M&H study, but such talk still comprised <2% of sampled conversations. Another potential problem with the EAR, participant self-selection bias, was addressed by comparing the EAR volunteers’ HEXACO personality dimensions to a non-volunteer sample drawn from the same student population. EAR volunteers were significantly and moderately higher in Conscientiousness, and lower in Emotionality, than non-volunteers. In conclusion, the EAR method can be successfully implemented in at least one all-party consent state (California). Interested researchers are encouraged to review this procedure with their own legal counsel.

## Introduction

Psychologists’ common reliance on (1) decontexualized behavioral responses to laboratory manipulations, and (2) global self-report instruments, limits the ecological validity of inferences about social processes and personality variation (Funder, 2001; Rozin, 2001). Growing recognition of this problem has inspired the development and evaluation of a variety of methods for systematically sampling human behavior outside the laboratory (Mehl and Conner, 2012; Robbins, in press). One such method, the Electronically Activated Recorder (EAR; Mehl et al., 2001; Mehl and Robbins, 2012; Mehl et al., personal communication) unobtrusively collects periodic brief audio snippets of people’s daily lives, using a portable recording device that participants wear attached to their clothes during their waking hours. Though the function of the EAR—to acoustically sample social environments—has remained consistent since 2001, the technology used to make the recordings has evolved. The EAR has progressed from digital tape recorders to the current iPod Touch devices using the “iEAR” app (Mehl, 2017). As technology has progressed, the legal landscape has also adapted to address the potential for others to be recorded on smartphones and other devices, such as iPods. Because of this, methods such as the EAR need to be adjusted to comply with laws (Robbins, in press). This study sought to examine how one such adjustment for the EAR—a button participants wear to notify others they may be recorded—may alter its obtrusiveness, participant compliance, and self-selection bias.

As a naturalistic, ecological method, the EAR resembles experience sampling methods (ESM: Csikszentmihalyi et al., 1977; Conner et al., 2009). The two methods differ, however, in the perspectives they capture (Mehl and Robbins, 2012). ESM participants are periodically prompted to report on their momentary cognitions, emotions, behaviors, and/or situations. Thus, ESM data represent the self’s subjective, experiential perspective, and therefore ESM methods are appropriate when research questions pertain to this perspective. ESM data are susceptible to some of the same limitations (e.g., impression management) as the more commonly used global or retrospective self-report instruments. In contrast, the EAR method captures a bystander’s perspective on behavior. A wide variety of behavioral variables, including speech content, can be reliably coded from EAR-generated recordings. Mehl and Pennebaker (2003) found substantial inter-rater reliability and within-participant temporal stability in variables pertaining to participant location, activity (e.g., amusement, attending lecture), social interaction (e.g., talking to others, laughing), and language use as measured by the Linguistic Inquiry and Word Count software (LIWC; Pennebaker et al., 2001). Self-reported major personality dimensions are correlated in predictable ways with some EAR-measured variables (e.g., Extraversion negatively correlated with percentage of time spent alone; Mehl et al., 2006).

In recent years, a number of other research questions have been addressed using EAR-generated data, usually in conjunction with self-report measures. These questions include the relative accuracy of self- and other-reports of daily behavior (Vazire and Mehl, 2008), debunking myths about sex differences in talkativeness (Mehl et al., 2007), individuals’ and couples’ means of coping with chronic illness (Robbins et al., 2011, 2014), interpersonal conflict and physical health (Tobin et al., 2015), and social behavior associated with borderline personality disorder (Tomko et al., 2012) and major depressive episodes (Baddeley et al., 2012). Importantly, in all these studies, data of comparable precision, free of the effects of inaccurate self-perception, impression management bias and faulty recall, could not have been collected using more conventional methods.

Methods, such as the EAR, that directly sample behavior during daily life raise legal and ethical issues that do not arise in laboratory or questionnaire studies. For example, wearable cameras can produce photographs at regular intervals, and these photographs can then be used as memory cues by participants as they segment their time into situations and describe them (Brown et al., 2017). This method requires privacy safeguards that closely parallel those practiced in EAR protocols (see The EAR Method: Details and Privacy Safeguards). As another example, smartphone apps have enormous potential to collect data on physical activity, physiological states, locations, and social networks (Miller, 2012). This general method raises legal and ethical issues that have barely been seriously considered.

### The EAR Method: Details and Privacy Safeguards

Mehl et al. (2012) describe EAR methodological practices. For a 2–4 days period, participants wear a recording device (currently, an iPod touch running a dedicated app, iEAR) attached to their clothing, using a protective case with a clip, during all their waking hours to the extent possible. The device makes periodic brief time-stamped audio recordings – in the most typical sampling pattern, a 30-s recording every 12.5 min. Participants know the general sampling pattern. However, the device is locked with a passcode, preventing participants from knowing when recording is occurring, to allow them to conduct their normal daily activities as much as possible. They are also asked to complete an hourly event diary at the end of the monitoring period, noting their major activities and times when they were not wearing the device, as an aid to researchers’ interpretations of the audio clips.

Participants’ privacy is protected by three safeguards. First, the aggregate recording time comprises only 5% of each participant’s day (using the most typical sampling rate), and the brief duration of the clips ensures that they do not capture very much contextualized personal information. Second, at the end of the recording period, participants in all EAR studies are given the opportunity to privately review their audio clips and to delete as many as they wish, before researchers listen to them. Across studies, the percentage of clips deleted is well under 1% (Mehl et al., 2012). Third, no recordings are made during an overnight blackout period (typically, 00:00 to 06:00).

### Legal and Methodological Issues

The EAR method raises both legal and methodological issues. The principal legal issue is the ban, in some jurisdictions, on recording conversations in the absence of the consent of all conversation participants. Applied to the EAR method, these laws require that participants’ interlocutors and other bystanders consent to the recordings. In contrast, photographs taken in public places by wearable cameras are not subject to bystander consent requirements (Brown et al., 2017). Eleven to thirteen US states (hereafter, “all-party consent states”; legal opinions differ on exactly how many fall in this category) require the consent of all parties to the recording of conversations (Reporters Committee for Freedom of the Press, 2012; Digital Media Law Project, 2014). Several non-US jurisdictions have similar laws ^1^, though it is not always clear whether they apply only to telephone conversations or to face-to-face conversations as well. Interested researchers are advised to consult local legal counsel before conducting an EAR study.

As described in detail below, our EAR participants displayed to potential interlocutors, a prominent visual text warning (“bystander button”) that their voices may be recorded to comply with California state law. In our view, and under the advice of our legal counsel, such a warning removes the reasonable expectation that the conversation is private. Thus, continuing the in-person exchange serves as passive consent to be recorded. However, this visual text warning also potentially exacerbates three of the EAR’s methodological problems. These problems are (1) participant non-compliance, (2) obtrusiveness or demand characteristics, and (3) self-selection bias. How frequently do participants actually keep the recording device close enough to generate valid recordings? To what extent does awareness of the recording device affect participants’ behavior? Does willingness to wear the bystander button introduce problematic self-selection bias to EAR participant samples? The first two questions have been addressed empirically (Mehl and Holleran, 2007; Robbins et al., 2014), but not using an EAR protocol that attempts to comply with the laws of an all-party consent state. As described in more detail below, research has shown that participants are compliant, on average, during 80–90% of their waking time. In debriefing surveys, they report that their behavior, and the behavior of their interlocutors, was only slightly affected by the presence of the recording device. Conversations about the recording device have comprised less than 4% of participants’ conversations. However, it is unknown to what degree these findings are applicable in an all-party consent jurisdiction like California.

### The Present Study

The first goal of the present study is to determine whether the prominent visual text warning, required in all-party consent jurisdictions, decreases EAR participant compliance and/or increases perceived obtrusiveness. To answer these questions, we compared measures of compliance and perceived obtrusiveness between a data set provided from a published EAR study in a one-party consent state (Mehl and Holleran, 2007), and a new EAR data set collected in California, which requires all-party consent to recording conversations where there is a reasonable expectation of privacy.

A third potential methodological problem of the EAR is participant self-selection bias. EAR participation is arguably more burdensome, both psychologically and logistically, than completing a self-report questionnaire or performing a laboratory task. Further, the requirement that participants wear a button visibly on their shirt that indicates conversations may be recorded could add to this burden. Do samples of EAR research volunteers differ from the broader populations from which they are drawn? If so, how? The second goal of the present study is to describe self-reported personality differences between (1) a sample of EAR volunteer participants, who were not drawn from a college course participant pool but were paid for their participation, and (2) a sample, recruited from the same student population, of people who did not volunteer for the EAR study but were only completing a self-report questionnaire as one means of fulfilling a course requirement.

We measured differences between the EAR sample and the comparison sample in the HEXACO personality dimensions (Ashton and Lee, 2007). Three of the HEXACO dimensions — Extraversion, Conscientiousness, and Openness — are quite similar to their counterparts in the more widely used Five Factor Model (FFM: McCrae and John, 1992). Two of the HEXACO dimensions, Agreeableness and Emotionality, represent an alternative rotation of the personality space covered in the FFM by Agreeableness and Neuroticism (Ashton and Lee, 2007). Two of the Emotionality facets, Anxiety and Fear, are also found in FFM Neuroticism, but its other two facets are Sentimentality and Emotional Dependence, which in some respects resemble facets of FFM Agreeableness. Unlike FFM Neuroticism, HEXACO Emotionality does not include Anger; instead, Anger is at the low pole of HEXACO Agreeableness. The HEXACO model’s sixth dimension, Honesty-Humility, is its most distinctive. Comprised of the facets Sincerity, Fairness, Greed Avoidance, and Modesty, Honesty-Humility is a better predictor than any FFM dimension of a wide variety of exploitative and anti-social behaviors (Lee and Ashton, 2005; Lee et al., 2013).

Two considerations suggest that EAR volunteers will differ from their non-volunteering counterparts with respect to one or more major dimensions of personality. First, successful EAR participation demands behaviors associated with Conscientiousness, e.g., temporarily taking responsibility for another party’s valuable possession (the iPod) and remembering to recharge the device nightly. Second, people with prosocial (as distinct from individualistic and competitive) social value orientations are generally more likely to volunteer for psychological experiments (Rosenthal and Rosnow, 1975; Van Lange et al., 2011). However, because participants in the current study received a substantial ($50) compensation, we did not predict that either of the two HEXACO dimensions related to prosociality (Honesty-Humility and Agreeableness — Ashton and Lee, 2007) would be higher in EAR volunteers than in non-volunteers. Comparisons with respect to the traits other than Conscientiousness should be regarded as exploratory analyses.

## Materials and Methods

### Participants, Procedures and Analyses: One-Party Consent Compliant EAR Study

M. Mehl (personal communication) provided to the authors the raw data on which the analyses reported by Mehl and Holleran (2007) were based. Their Sample 1 consisted of 96 undergraduates (49% female, *M* age = 18.7, *SD* = 0.9) who wore the EAR for 48 h on weekdays. They were then asked to complete the EAR evaluation questionnaire, which consists of eight items, with five-point Likert-style response scales, tapping the obtrusiveness of EAR participation (**Table 1**). They were also asked to estimate the percentage of their waking time during which they were not wearing the EAR (self-reported compliance). In addition, two behavioral variables were coded from the EAR recordings. As a measure of obtrusiveness, a percentage of each participant’s recorded conversations that included mention of the EAR study was calculated. As a measure of compliance, the percentage of audio clips during which the participant was judged not to be wearing the device (i.e., non-compliant) was calculated, based on auditory characteristics. For more details about this study’s sample and procedures, see Mehl et al. (2006) and Mehl and Holleran (2007).

**Table 1:** Self-reported and behaviorally assessed EAR obstrusiveness and compliance in the all-party consent compliant study (the present study), and comparison with the one-party consent compliant study of Mehl and Holleran (2007).

Measure	*M*	*SD*	Difference from Mehl and Holleran Sample 1	*t*	*p*	Cohen’s *d*
Self-reported obtrusiveness for participants: To what degree…						
1. …were you generally aware of the EAR?	3.0	0.9	0.1	0.34	0.73	+0.06
2. …did you feel uncomfortable wearing the EAR?	1.8	0.9	–0.2	–1.00	0.32	–0.16
3. …did the EAR impede you in your daily activities?	1.4	0.5	–0.4	–3.48	0.0006	–0.56
4. …did the EAR change your actual behavior?	1.4	0.6	–0.2	–2.07	0.04	–0.32
5. …did the microphone influence your way of talking?	1.3	0.6	–0.3	–2.35	0.02	–0.38
Scale	1.8	0.4	–0.2	–2.30	0.02	–0.30
Self-reported obtrusiveness for bystanders: To what degree…						
6. …were people around you aware of the EAR?	3.0	1.1	–0.2	–1.34	0.16	–0.22
7. …did you talk to people around you about the EAR?	3.2	0.9	–0.4	–2.32	0.02	–0.37
8. …did the EAR influence the behavior of people around you?	1.8	0.8	–0.2	–1.32	0.19	–0.21
Scale	2.7	0.7	–0.3	–2.17	0.03	–0.34
Behaviorally assessed obtrusiveness						
Percent of conversations about the EAR	1.9	3.0	0.6	1.99	0.05	0.30
Self-reported compliance						
Percent of time awake not wearing the EAR	13.9	9.9	–8.6	–3.93	0.0001	–0.64
Behaviorally assessed compliance						
Percent of time not wearing the EAR	8.1	8.6	1.1	–0.58	0.56	–0.09

*N = 72. Items 1–8 used a scale of 1 = “Not at all” to 5 = “A great deal”.*

*Reproduced with permission from Mehl and Holleran (2007).*

### Participants and Procedures: All-Party Consent Compliant EAR Study

This study will be referred to in this paper as “the present study.” Ninety-six students at the University of California, Los Angeles (UCLA), were recruited through class announcements and posted flyers. They were not drawn from a course participant pool (i.e., their participation was entirely voluntary). The public title of the study was “Audio Sampling of Daily Life.” Participants were briefed and issued an iPod programmed with the iEAR app. Compensation in the form of a $50 Amazon gift card was offered for completing the study. At their first appointments, participants were briefed using scripts slightly modified from those in the iEAR Researcher’s Guide (Mehl et al., personal communication), which encourages establishing rapport with participants (e.g., by assuring them that their privacy will be respected and encouraging them to review their audio clips and delete any clips they wish). The recording period lasted 72 h, during which one 30-s recording was made every 12.5 min, except between 00:00 and 06:00, when no recordings occurred. Recording periods could start on any weekday. Participants were also instructed to keep an hour-by-hour event diary at the end of every day, in which they noted their general activity and whether they were wearing the iPod during each hour between 6:00am and midnight. The study’s theory-driven hypotheses, which were drawn from Life History Theory, are beyond the scope of this paper (Manson, 2017).

Compliance with California’s all-party consent law (California Penal Code §632) was implemented using a method reviewed by an in-house attorney for the University of California, Riverside (Robbins, in press). Participants were told about the law and were given a 5.5 cm diameter button, bearing a microphone icon and the text, “This conversation may be recorded.” Materials for this procedure can be found online at the EAR Repository (Robbins et al., 2016) ^2^. They were instructed to wear this button on the front of their clothing at all times while they were wearing the EAR. It is important to note that this solution must be reviewed by researchers’ own legal counsel before implementing it as a solution to legally implementing an EAR study in an all-party consent jurisdiction.

At their second appointments, participants returned the iPod. While the experimenter was uploading the sound files to a laptop computer, participants were asked to complete the EAR evaluation questionnaire (see EAR Evaluation Questionnaire). After the upload was complete, participants were given the opportunity to review and delete as many audio clips as they wished. They were provided with headphones, and they were encouraged to refer to their own completed event diaries to help them focus on time periods that might be of particular concern to them. Finally, participants were given a link and a password to an online SurveyMonkey^TM^ survey which contained several self-report instruments, including the HEXACO-60 (Ashton and Lee, 2009). After completing the self-report instruments, participants were sent the code with which to access their compensation.

### Participants and Procedures: Non-EAR Comparison Sample

One hundred and sixty-two UCLA students (102 female, 16 of unknown gender as a result of a programming error, *M* age = 19.2, *SD* = 1.6) completed several self-report instruments, including the HEXACO-60, in fulfillment of a research participation requirement in an introductory Communication Studies course. The study title provided in the recruitment script was “Personality and Life Experiences Survey.” Participants were pre-screened to ensure that none of them were participants in the EAR study. The self-described ethnic composition of non-EAR UCLA comparisons sample was 31.5% White, 30.9% Asian or Asian-American, 19.1% Latino/a, 3.7% African or African-American, 1.8% Middle Eastern, and 13% mixed, “other,” or “decline to state.” Although unrepresentative of college-aged Americans generally, the sample’s ethnic composition was fairly representative of the UCLA undergraduate student body. As of Fall 2014, this was reported to be 33.5% Asian/Pacific Islander, 12.6% International, 27.1% White, 19.1% Hispanic, 4.0% African-American, 0.5% Native American, and 3.1% of Unknown race/ethnicity^3^.

### Self-report Measures

#### EAR Evaluation Questionnaire

The EAR evaluation questionnaire (**Table 1**) was the same as that used by Mehl and Holleran (2007). Participants were also asked to (1) rate how typical, on a five-point scale (1 = “not at all” to 5 = “a great deal”), the 72-h recording period had been with respect to their usual activities, (2) briefly describe how, if at all, it had been atypical and (3) share any thoughts and feelings they wished about the experience of wearing the EAR.

#### HEXACO-60

The HEXACO-60 (Ashton and Lee, 2009) is a shorter version of the HEXACO Personality Inventory—Revised (Lee and Ashton, 2004). This instrument contains 10 items tapping each of the six HEXACO dimensions: Honesty-Humility, Emotionality, Extraversion, Agreeableness, Conscientiousness, and Openness to Experience. Participants responded on a five-point scale.

### EAR Behavior Coding

In addition to the self-report obtrusiveness and compliance items, two behavioral variables were coded from the EAR recordings, following Mehl and Holleran (2007). All intelligible speech by participants was transcribed. As a behavioral measure of obtrusiveness, a percentage of each participant’s recorded conversations that included mention of the EAR study was calculated. As a behavioral measure of compliance, the percentage of audio clips during which the participant was judged not to be wearing the device (i.e., non-compliant) was calculated, based on auditory characteristics in combination with the entries in the participant’s hour-by-hour event diary. For example, some clips were coded as non-compliant even when the event diary indicated compliance for that time period, based on inconsistency between the two information sources (e.g., an event diary entry of “Dinner in dining hall” in conjunction with clips containing only low-amplitude ambient noise, indicating that the participant left the device in her dorm room while she went to dinner).

### Data Analysis

Each participant’s clips were coded by one coder. The inter-rater reliabilities of the two behavior codes, along with all the other behavior codes used in the broader research project, were assessed by assigning the 11 research assistants to code a test set of 110 clips containing at least one exemplar of every coded behavior category. Research assistants coded these clips independently, and inter-rater reliability was measured with Cohen’s κ.

Each HEXACO dimension is comprised of four facets (Ashton and Lee, 2007), each of which is tapped by 2–3 items of the HEXACO-60 (Ashton and Lee, 2009). Each facet score was calculated as the mean score across its constituent non-missing items. Each dimension score was then calculated as the mean across its four facet scores.

We used *t*-tests and effect size calculations (Cohen’s *d*) to compare results from the present study to Mehl and Holleran’s results, and to compare HEXACO dimension scores between our EAR and non-EAR participants. For the data from the present study, we also examined gender differences in perceived obtrusiveness. The ethnic diversity of the present study’s sample (see Descriptive Results: Present Study) limited the statistical power of potential tests of ethnic differences in perceived obstrusiveness. We carried out one such test, comparing Asian and Asian-American participants to other participants. All statistical tests are two-tailed.

## Results

### Descriptive Results: Present Study

Of the 96 EAR participants, 72 (56.9% female, *M* age = 20.3, *SD* = 3.3) completed EAR participation, the EAR evaluation questionnaire, and the HEXACO-60. The self-described ethnic composition of this sample was 53.5% Asian or Asian-American, 16.9% White, 11.2% Latino/a, 2.8% Middle Eastern, and 15.5% mixed or “other”.

Eight participants did not complete the study because of their own non-compliance (e.g., neglecting to recharge the EAR), 10 participants did not provide useable EAR recordings because of technical problems (six of which were traced to a problem with the internal microphone of one iPod), and four participants did not provide useable EAR recordings because of experimenter error. One participant completed the EAR monitoring but did not complete the online self-report questionnaires. One of the participants who completed both the EAR recordings and the online self-report questionnaires did not complete the hard copy EAR evaluation questionnaire. The mean (± SD) percentage of audio clips deleted by participants was 1.1% (± 2.7%), with a median of zero and a maximum of 17.6%. Expressed as raw numbers of clips, participants deleted an average of 2.8 (± 6.5) clips, with a maximum of 39.

**Table 1** shows the mean scores and standard deviations for the EAR evaluation questionnaire items and the behavioral measures of obtrusiveness and compliance in the present study. The highest means were for the two items (6 and 7) that reveal participants did not hide the device from others, and in fact, introduced it, which suggests compliance with our legal and ethical procedures. Also shown in **Table 1** are the means and standard deviations of the two self-report obtrusiveness subscales: obtrusiveness for participants (mean of items 1–5) and obtrusiveness for bystanders (mean of items 6–8). The internal reliability of these subscales were α = 0.59 and α = 0.61, respectively. In response to the open-ended prompt to reflect on the experience of wearing the EAR, only 6 of the 72 participants (8.3%) mentioned negative reactions to the button warning about the recordings. These accounts referred to friends and colleagues expressing feelings of disapproval, discomfort, or self-consciousness, and/or to strangers’ suspiciousness, stares, and unwanted questions. Participants were instructed to pause the device or remove it in instances where conversation partners were uncomfortable being recorded; however, they did not report how they resolved these encounters.

Perceived obtrusiveness did not vary by participant gender. Mean (± SD) obtrusiveness for participants was 1.78 ± 0.46 among women (*n* = 41), and 1.75 ± 0.40 among men (*n* = 31) (*t* = -0.20, *d* = 0.07). Mean (± SD) obtrusiveness for bystanders was 2.65 ± 0.58 among women, and 2.75 ± 0.84 among men (*t* = 0.61, *d* = 0.14). Sample size limited our ability to examine potential ethnic differences in perceived obtrusiveness, but the most powerful possible analysis (Asians and Asian-Americans, *n* = 38, compared to all others, *n* = 34) revealed no significant differences (obtrusiveness for participants: Asians and Asian-Americans, *M* ±*SD* = 1.76 ± 0.49, others, *M* ±*SD* = 1.77 ± 0.37, *t* = 0.07, *d* = 0.02; obtrusiveness for others, Asians and Asian-Americans, *M* ±*SD* = 2.70 ± 0.68, others, *M* ±*SD* = 2.69 ± 0.73, *t* = -0.09, *d* = 0.01).

Among 11 coders of the behavioral measures, inter-rater reliability as measured by Cohen’s κ was substantial for both compliance (κ = 0.72) and conversational mention of the EAR study (κ = 0.82). The correlation between behaviorally assessed compliance and self-reported compliance was 0.29 (*p* = 0.014).

### Differences between Studies in Compliance and Obtrusiveness

**Table 1** also shows, for each item, the difference between the scores from the present study and the scores from Mehl and Holleran’s (2007) non-all-party consent compliant study, as well as statistical comparisons (*t*-tests and Cohen’s *d*). Participants generally experienced EAR participation as *less* obtrusive, both to themselves and their interlocutors, in the present study compared to Mehl and Holleran’s (2007) study, even though the present study’s participants were required to wear the bystander button. For example, participants reported that the EAR impeded their daily activities and changed their behavior significantly less than in the Mehl and Holleran (2007) study. Effect sizes of these differences were generally small. Participants in the present study talked more about being in the study than did participants in Mehl and Holleran’s (2007) study, with a small effect size. Self-reported compliance was higher in the present study than in Mehl and Holleran’s (2007) study, but behaviorally assessed compliance did not differ between the two studies.

### HEXACO Dimension Scores in the EAR and Non-EAR Comparison Samples

Among the 73 EAR volunteers who completed the HEXACO-60 (including the one participant who completed the EAR protocol but not the EAR evaluation questionnaire), 21/4380 (0.48%) responses were missing. Among the 162 participants of the non-EAR comparison sample, 39/9720 (0.40%) responses were missing. **Table 2** shows, for each HEXACO dimension, Cronbach’s α for the two samples and the results of *t*-tests and the effect sizes of the difference between the samples. Conscientiousness was higher, and Emotionality was lower, in the EAR volunteers than in the non-volunteers. The effect sizes of both differences were in the small to moderate range. None of the other HEXACO dimensions (Extraversion, Agreeableness, and Openness) differed between the samples, although there was a non-significant trend (*p* = 0.06) for EAR volunteers to score higher than non-volunteers on Honesty-Humility.

**Table 2:** HEXACO dimension scale reliability and scores of EAR volunteers and non-volunteer comparison sample.

	Honesty-Humility		Emotionality		Extraversion		Agreeableness		Conscientiousness		Openness	
	α	*M* (*SD*)	α	*M* (*SD*)	α	*M* (*SD*)	α	*M* (*SD*)	α	*M* (*SD*)	α	*M* (*SD*)
EAR volunteers	0.75	3.22 (0.65)	0.67	3.29 (0.59)	0.82	3.37 (0.71)	0.84	3.12 (0.75)	0.70	3.70 (0.50)	0.71	3.62 (0.62)
Non-volunteers	0.72	3.06 (0.60)	0.74	3.48 (0.62)	0.81	3.40 (0.67)	0.76	3.14 (0.60)	0.76	3.49 (0.61)	0.74	3.49 (0.60)
*t*	1.90		–2.21^∗^		–0.38		–0.23		2.67^∗∗^		1.49	
Cohen’s *d*	0.26		–0.31		–0.06		–0.03		0.38		0.21	

*Dimension scores were calculated as the mean across each dimension’s four facet scores. Each facet scores were calculated as the mean across its constituent non-missing items. ^∗^*p* < 0.05; ^∗∗^*p* < 0.01.*

As described in Sections “Participants and Procedures: Non-EAR Comparison Sample and Descriptive Results: Present Study,” the ethnic composition of the EAR sample differed from that of the non-EAR comparison sample. Most notably, the EAR sample was 53.5% Asian or Asian-American, whereas the non-EAR comparison sample was 30.9% Asian or Asian-American. However, the differences in Conscientiousness and Emotionality between the two samples cannot be attributed to their differences with respect to proportion of Asians and Asian-Americans. Removing the data from Asian and Asian-American participants from both samples produced little change in the group means or effect size for Conscientiousness (EAR: 3.73 ± 0.50; non-EAR: 3.53 ± 0.63; *d* = 0.35). For Emotionality, the difference between the EAR and non-EAR samples was increased by removing from analysis the data from Asian and Asian-American participants (EAR: 3.23 ± 0.57; non-EAR: 3.51 ± 0.63; *d* = 0.47).

## Discussion

This study examined the obtrusiveness, participant compliance, and self-selection bias of an EAR protocol adjusted to comply with an all-party consent recording law, compared to the original EAR protocol. Wearing a button bearing the words “This conversation may be recorded” did not increase the method’s self-reported obtrusiveness to self or others, compared to a published data set collected from participants who did not wear the button (Mehl and Holleran, 2007). Further, compliance—participants’ reported and behaviorally assessed wearing the EAR—was highly comparable to the original protocol. Lastly, we found that self-selection bias for those who volunteer for our EAR study versus a non-volunteer sample differed mostly in ways common to all volunteer samples. In sum, these results provide evidence that the EAR method is feasible in an all-party consent jurisdiction such as California.

Self-reported obtrusiveness was significantly lower in the study requiring the bystander button. It is unclear whether this is attributable to sampling error, cultural changes surrounding the prevalence of smartphones and their recording functions during the approximately 10 years separating the two studies, or the difference between sample sources [students fulfilling a course requirement in the Mehl and Holleran (2007) study vs. students offered monetary compensation in the present study]. In any case, the results indicate that participants in an all-party consent state, at minimum, do not perceive the EAR method with the bystander button as more obtrusive than it is in a one-party consent state without use of the button. Perceived obtrusiveness did not vary by gender or (to the extent that we could test for differences) self-identified ethnicity.

Behaviorally assessed obtrusiveness (proportion of conversations in which participants discussed the EAR study) was significantly higher in the present study than in the Mehl and Holleran (2007) study, but was still low (<2% of conversations). This finding is particularly important, as it indicates that the bystander button is effective at increasing awareness that recording is taking place. In conjunction with the finding that the button did not increase perceived obtrusiveness of the method, this indicates that the bystander button serves its main purpose of alerting others to the recording, while doing so with minimal disruption to participants’ daily lives.

Further, rates of behaviorally assessed non-compliance with wearing the EAR device did not differ between the present study and the Mehl and Holleran (2007) study, while self-reported non-compliance was significantly lower in the present study. It is important to note that behavioral non-compliance is the most important measure of this construct, as it reveals how much usable EAR data a study has yielded.

A limitation of the present study is that participants’ interlocutors could not be queried directly regarding their perception of the EAR protocol or how it affected their behavior. Furthermore, potential interlocutors, who did not already know participants, might have avoided interacting with them upon seeing the bystander button. Thus, the all-party consent compliance protocol of this study could have altered participants’ social micro-environments in ways that escaped their awareness. Another limitation of the present study is that the participants, and most of their interlocutors, were college-aged. Some research (Hoofnagle et al., 2010) indicates that younger adults are less concerned about online privacy than older adults. Perhaps this difference generalizes to concern about the privacy of face-to-face conversations. If so, then older participant samples would be expected to show higher levels of perceived obtrusiveness in an EAR study using the bystander button.

As predicted, volunteers for an EAR study were more Conscientious than a sample of non-volunteers drawn from the same student population. This is consistent with past work revealing characteristics typical of people who volunteer for psychological studies in general (Rosenthal and Rosnow, 1975; Van Lange et al., 2011). Nevertheless, in any EAR study that includes Conscientiousness, or a trait that is strongly correlated with it, as an independent variable of interest, range restriction could reduce the power of the study to detect hypothesized relationships. Among the other HEXACO dimensions, only Emotionality differed between the EAR volunteer and non-volunteer samples. One of the Emotionality facets is Anxiety, and it is plausible that college students who are more susceptible to Anxiety are more reluctant, even when assured of confidentiality, to expose an audio record of their everyday behavior to examination by a research team. The HEXACO-60 includes only two items that tap the Anxiety facet. Reliability among these items was low (α = 0.42), so the present data set does not permit a test of whether Anxiety, specifically, differed between the EAR volunteer and non-volunteer samples.

## Conclusion

The EAR method can be successfully implemented in at least one all-party consent state. Researchers wishing to use the EAR method in all-party consent jurisdictions should not be discouraged from doing so by the requirements of complying with legal restrictions; rather, they should work with their own legal and ethical counsel to adopt this or another solution for implementing an EAR study. The specifics of all-party consent laws vary across jurisdictions, and ethical concerns with this method may vary across IRBs. We encourage researchers to review the procedure described here with their own legal counsel at their university to ensure proper compliance.

## Ethics Statement

The present study was approved by the Institutional Review Board of the University of California, Los Angeles (Approval #12-001128). Written informed consent was obtained from all participants in accordance with the terms of that approval.

## Author Contributions

MR developed the bystander button protocol and provided extensive comments and edits on the manuscript. JM designed the present study, collected the data, analyzed the results, and wrote the first draft of the manuscript.

## Conflict of Interest Statement

The authors declare that the research was conducted in the absence of any commercial or financial relationships that could be construed as a potential conflict of interest.

## Footnotes

1 https://en.wikipedia.org/wiki/Telephone_recording_laws

2 osf.io/n2ufd

3 http://www.aim.ucla.edu/profiles/main.pdf
